# It Looks Like a Zebra but Is Not: [^18^F]FDG PET/CT in a Giant Cutaneous Malignant Melanoma Mimicking Squamous Cell Carcinoma

**DOI:** 10.3390/diagnostics14242860

**Published:** 2024-12-19

**Authors:** Ilaria Proietti, Giulia Azzella, Diana Dirzu, Claudio Di Cristofano, Oreste Bagni, Concetta Potenza, Luca Filippi

**Affiliations:** 1 Dermatology Unit “Daniele Innocenzi”, “A. Fiorini” Hospital, Via Firenze, 1, 04019 Terracina, Italy; ilaria.proietti@uniroma1.it (I.P.); azzellagiulia1996@gmail.com (G.A.); concetta.potenza@uniroma1.it (C.P.); 2 Department of Dermatology, Grigore T. Popa University of Medicine and Pharmacy, Railway Clinical Hospital, 700506 Iasi, Romania; dirzudianastefania@gmail.com; 3 Department of Medical-Surgical Sciences and Bio-Technologies, Sapienza University of Rome, 04100 Latina, Italy; claudio.dicristofano@uniroma1.it; 4 Department of Nuclear Medicine, Santa Maria Goretti Hospital, AUSL Latina, 04100 Latina, Italy; o.bagni@ausl.latina.it; 5 Department of Biomedicine and Prevention, University of Rome “Tor Vergata”, Via Montpellier 1, 00133 Rome, Italy

**Keywords:** cutaneous tumor, malignant melanoma, PET/CT, targeted therapy, metabolic imaging, precision medicine

## Abstract

Cutaneous malignant melanoma (MM) is the most aggressive form of skin cancer, associated with high mortality and rising incidence rates in Europe despite prevention efforts. Nodular MM, the most aggressive subtype, often mimics other skin tumors, complicating diagnosis. We present the case of a 66-year-old woman with a large, ulcerated tumor beneath the left scapula, along with multiple nodular lesions on the left arm and chest. Initially suspected to be an aggressive squamous cell carcinoma, the diagnosis was confirmed as invasive cutaneous MM with a BRAF(V600) mutation via biopsy. Staging with PET/CT revealed extensive glucose metabolism in the tumors and surrounding tissues, as well as metastatic lymphadenopathy. The disease was classified as stage IV (T4bN3cM1a0). Neoadjuvant systemic therapy with BRAF and MEK inhibitors (Dabrafenib and Trametinib) was initiated to reduce tumor size. Remarkable regression was observed within a week, with further reduction in tumor size after one month. A follow-up PET/CT after 3 months showed significant decreases in tracer uptake and lesion size, with a ΔSUVmax of 51.9%, a ΔMTV of 74.5%, and a ΔTLG of 83.5%, indicating an excellent response to targeted therapy.

**Figure 1:**
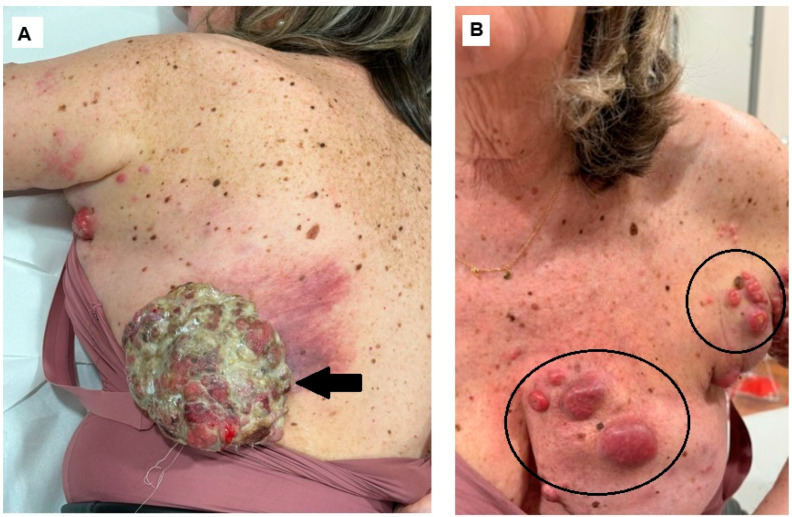
Cutaneous malignant melanoma (MM) is the most aggressive form of skin cancer, associated with a high mortality rate and accounting for over 80% of skin cancer deaths [1]. In Europe, despite the implementation of primary and secondary prevention methods, its incidence continues to rise [2]. Nodular MM is the most aggressive subtype of MM, and its clinical presentation can sometimes be misleading due to its resemblance to other skin tumors such as seborrheic keratosis, squamous cell carcinoma, nodular basal cell carcinoma, or pyogenic granuloma [3,4,5]. Here, we present the case of a 66-year-old woman who was referred to our clinic with a large, round, ulcerated tumor mass measuring 13 cm in diameter located beneath the left scapula along with multiple nodular skin lesions of varying sizes on the left arm and left side of the chest. ((**A**), arrow) Voluminous cutaneous tumor in the left scapula; ((**B**), circles) multiple nodular skin lesions of varying sizes on the left arm and left side of the chest: specifically, in the upper lateral and medial quadrants of the breast and in the axilla. The patient reported that the lesions appeared two years ago and have been growing progressively since then. In recent months, the primary lesion has enlarged significantly, and satellite lesions have begun to emerge. During physical examination, we observed multiple enlarged lymph nodes in the left axilla, which were fixed to the surrounding soft tissue. Based on the clinical characteristics, an aggressive form of squamous cell carcinoma with satellitosis was initially hypothesized. She was submitted to incisional biopsy, positive for ulcerated MM, positive for BRAF(V600) mutation.

**Figure 2:**
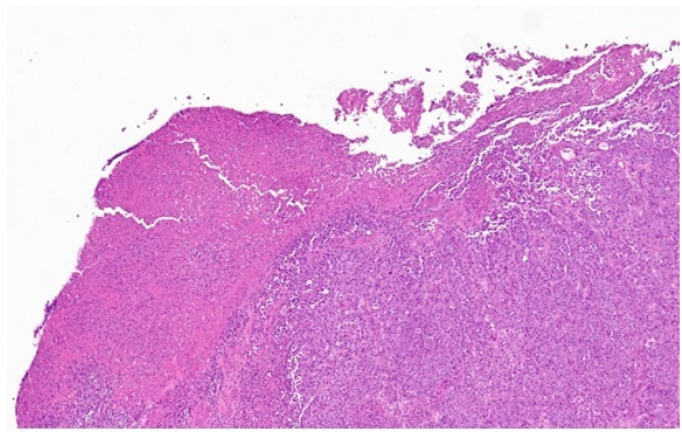
A tissue fragment measuring 1.5 × 1 × 0.3 cm was excised. Microscopic examination revealed a neoplasm composed of atypical melanocytes, organized in nests and solid cords, with a proliferative index (Ki67) of 20% and positivity for immunohistochemical markers typical of melanoma (S100+, MelanA+, HMB45+, and panCKAE1/AE3-) [6]. Due to the limited size of the sample, the Clark level and Breslow thickness could not be determined. A positron mission computed tomography (PET/CT) with ^18^F-fluoro-deoxyglucose ([^18^F]-FDG) was requested as a staging procedure.

**Figure 3:**
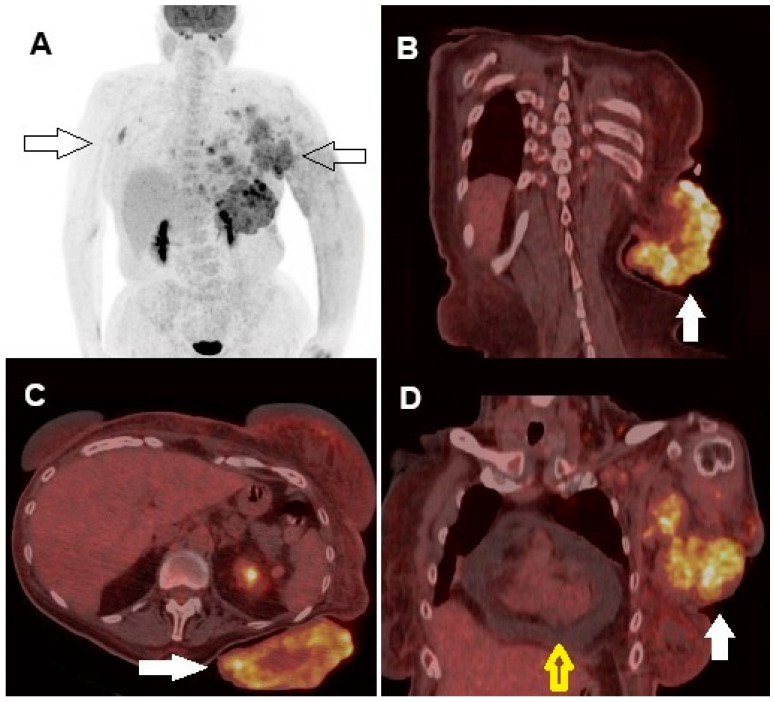
Whole-body [^18^F]FDG PET/CT showed increased glucose metabolism in the cutaneous and subcutaneous tissue surrounding the tumors, as well as in several lymph node stations (right axillary lymph nodes and, on the left side, axillary, supraclavicular, and along the internal mammary chain) ((**A**), arrows). High tracer uptake in the cutaneous lesion of the left scapula was clearly visible in the fused coronal ((**B**), arrow) and axial ((**C**), arrow) views, as well as in the fused coronal slice of the left breast region ((**D**), arrow). Furthermore, coronal images showed pericardial effusion ((**D**), yellow bordered arrow). Quantitative PET-derived parameters for the largest scapular region were as follows: maximum standardized uptake value (SUVmax), 10.4; metabolic tumor volume (MTV), 52 cc; and total lesion glycolysis (TLG), 267 g/mL. No visceral or skeletal metastases were detected. Subsequent cerebral magnetic resonance imaging (MRI) did not identify any brain metastases. According to the 8th edition of the AJCC TNM Classification, the disease stage was determined to be T4bN3cM1a0, corresponding to stage IV [7]. Given the locally advanced and large primary tumor along with the presence of multiple metastatic lymphadenopathies, we opted for neoadjuvant systemic treatment. Our aim was to reduce the tumor size to make resection feasible, thereby potentially improving the patient’s quality of life. The patient underwent combined targeted therapy with BRAF and MEK inhibitors: Dabrafenib 150 mg b.i.d. plus Trametinib 2 mg q.d. After only one week, the outcome was remarkable, with partial regression of both primary tumor and metastases, presence of necrotic areas, and size reduction, confirmed by further regression of the lesions upon objective evaluation at 1 month.

**Figure 4:**
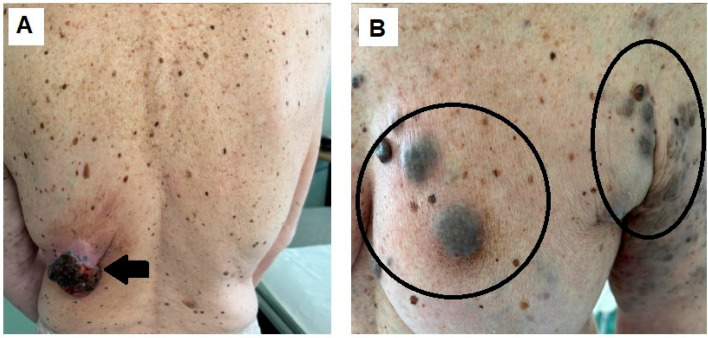
After 1 month of treatment, the primary tumor significantly shrank, reaching 5 cm in diameter ((**A**), arrow) as well the other localizations in the left breast region ((**B**), circles).

**Figure 5:**
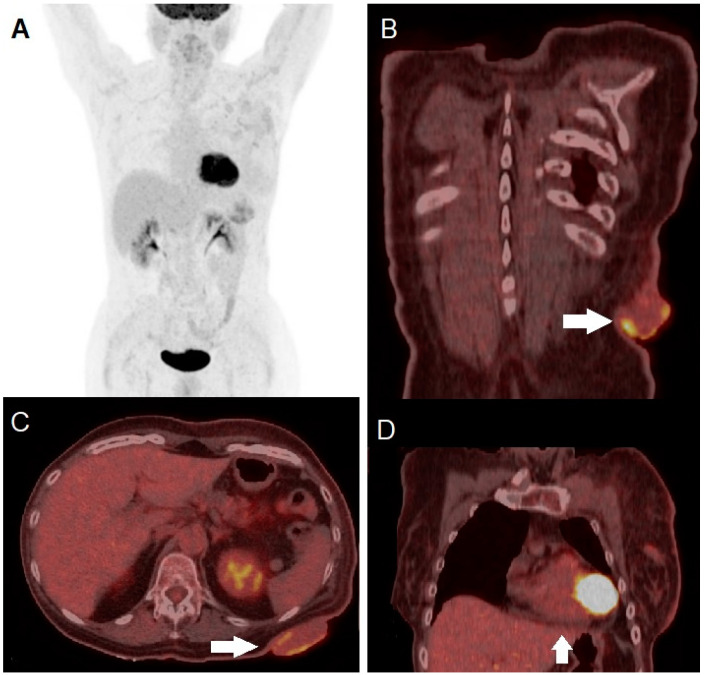
Follow-up PET/CT scan after 3 months, confirming response to treatment. Whole-body PET/CT showed a marked regression in the previously described foci of increased tracer uptake (**A**). Fused axial coronal ((**B**), arrow) and axial ((**C**), arrow) images demonstrated a significant reduction in tracer uptake and lesion size, clearly visible on both visual and quantitative analysis (SUVmax: 5, MTV: 14 cc, TLG: 44 g/mL), with a ΔSUVmax of 51.9%, a ΔMTV of 74.5%, and a ΔTLG of 83.5%. In addition, the fused coronal image demonstrated regression of the hypermetabolic lesions in the left breast region, as well as disappearance of the pericardial effusion ((**D**), arrow). After an additional three months, the patient underwent excision of the skin tumor in the left scapular region. Six additional months have now passed since the surgical excision, and the patient continues to be under active follow-up. The physical examination of the post-surgical scar has not shown any macroscopic signs of loco-regional recurrence so far, supporting the radicality of the intervention. She remains on molecularly targeted therapy, which is currently well tolerated. Despite the relatively short follow-up period, no signs of resistance to treatment have emerged to date. While we acknowledge the limitation related to the duration of follow-up, we believe this case highlights the potential of BRAF/MEK-targeted therapy in the neoadjuvant setting for managing a particularly large and aggressive melanoma. Cutaneous MM remains one of the most aggressive forms of skin cancer, with high rates of metastasis and poor prognosis, particularly in advanced stages [8]. The complexity in diagnosing and managing MM stems from its highly variable clinical presentation, which can often be misleading. Nodular melanoma, a notably aggressive subtype, is frequently mistaken for other skin lesions, such as seborrheic keratosis, basal cell carcinoma, Merkel cell carcinoma, or pyogenic granulomas [9]. This diagnostic challenge underscores the value of advanced imaging techniques in enhancing diagnostic accuracy and informing effective disease management [10]. Giant nodular melanomas are exceedingly rare but represent significant clinical and surgical challenges due to their destructive nature and poor prognosis [11]. Despite their high metastatic potential, a few cases in the literature have shown unexpectedly benign courses. To our knowledge, this is the first reported case of a metastatic giant cutaneous melanoma demonstrating an early remarkable response after targeted treatment, assessed both clinically and by [^18^F]FDG PET/CT scan. In a similar case described by Kruijff et al., a patient with stage IV cutaneous melanoma (T4N2M1c1) underwent palliative surgery, but despite intervention, the outcome was fatal within 12 weeks [12]. With the introduction of immunotherapies such as Ipilimumab in 2011, followed by PD-1, BRAF, and MEK inhibitors, long-term survival prospects for melanoma patients have improved significantly [13]. Adjuvant therapy has become the standard approach in advanced melanoma cases [14]. However, in our case, the rapid clinical response highlights the potential of targeted therapy as a primary approach. Monitoring treatment efficacy through visible changes in cutaneous lesions has provided critical insights into disease progression and response to therapy. Additionally, the atypical presentation of MM in this case—mimicking squamous cell carcinoma—emphasizes the need to consider a broad spectrum of differential diagnoses, encouraging vigilance among healthcare providers when confronted with unusual clinical features. This also reinforces the importance of personalized treatment approaches and showcases the efficacy of targeted therapies as neoadjuvant treatments in advanced MM management. Neoadjuvant therapy is a promising approach for patients with advanced melanoma [15,16]. However, due to a lack of randomized clinical trials definitively proving its superiority over traditional surgery with postoperative adjuvant treatment, neoadjuvant therapies have not yet become standard practice. A recently published study showed the potential of Dabrafenib plus Vemurafenib as neoadjuvant therapy, reporting a high rate of complete pathological responses (around 50%) and sustained disease control post-surgery, with relapse rates of around 17% [16]. The effectiveness of neoadjuvant therapy may be attributed to heightened immune system activity in response to a large primary tumor, promoting a stronger tumor-specific immune response. Excision of the primary tumor may subsequently alter its microenvironment, possibly diminishing the local immune response. However, it should be underlined that, while the rapid and impressive therapeutic response observed may suggest a potential contribution of immune modulation, it is important to note that, in the context of BRAF/MEK-targeted therapy, the role of the tumor microenvironment remains less well defined and less certain compared to its established importance in immunotherapy, though emerging evidence indicates it could play a supportive role [17]. In our case, imaging studies, particularly [^18^F]FDG PET/CT, were essential for an accurate assessment of disease extent. We opted for [^18^F]FDG PET/CT over total-body CT in our case due to its superior sensitivity and specificity in detecting non-pulmonary metastases in advanced melanoma [18]. This imaging modality provides enhanced metabolic imaging to complement anatomical details, which is particularly valuable in a setting where early and accurate staging can significantly influence therapeutic decisions. Moreover, [^18^F]FDG PET/CT entails a lower radiation burden than total-body contrast-enhanced CT, which is particularly relevant in patients requiring repeated imaging during treatment and follow-up. In our patient, the PET scan revealed elevated glucose metabolism in the primary tumor and in multiple regional lymph nodes, confirming metastatic disease. This finding significantly influenced our decision to initiate neoadjuvant therapy, aiming to reduce tumor size and improve surgical outcomes. PET/CT imaging is invaluable for staging and prognosis in melanoma patients [19,20]. Its ability to assess metabolic activity and identify regions of increased glucose uptake is critical for determining disease extent and informing accurate staging. This is particularly relevant in cases like ours, where clinical examination alone may not fully reveal metastatic spread. For instance, in our patient, [^18^F]FDG PET/CT revealed both the primary tumor and metastatic involvement in multiple lymph node stations, guiding our treatment strategy. Moreover, PET imaging serves as a powerful prognostic tool. Recent studies have indicated that percentage changes, the so-called “delta” (Δ), in PET-derived quantitative parameters (e.g., SUVmax, MTV, and TLG) correlate strongly with patient outcome in several tumors, including MM [21,22]. In our case, the significant Δ values obtained after 3 months of neoadjuvant therapy correlated significantly with the patient’s clinical objectivity and support the utility of PET imaging and PET-derived quantitative parameters in the more aggressive cases of melanoma, not only for staging but also for the early follow-up and prognostic stratification of patients.

## Data Availability

Not applicable.
